# Cloning, expression and characterization of l-asparaginase from *Pseudomonas fluorescens* for large scale production in *E. coli* BL21

**DOI:** 10.1007/s13205-015-0300-y

**Published:** 2015-04-05

**Authors:** Vijay Kishore, K. P. Nishita, H. K. Manonmani

**Affiliations:** Department of Biotechnology, Sapthagiri College of Engineering, Bangalore, 560 057 India; Fermentation Technology and Bioengineering Department, Central Food Technological Research Institute (CSIR), Mysore, 570 020 India

**Keywords:** l-Asparaginase, Cloning, *E. coli*, Enzyme activity

## Abstract

l-Asparaginase (E.C. 3.5.1.1) is used as an anti-neoplastic drug in the treatment of acute lymphoblastic leukemia. l-Asparaginase from *Pseudomonas fluorescens* was cloned and overexpressed in *E. coli* BL21. The Enzyme was found to be a Fusion protein-asparaginase complex which was given a lysozyme treatment and sonication, and then was purified in a Sepharose 6B column. The enzymatic properties of the recombinant enzyme were studied and the kinetic parameters were determined with kilometre of 109.99 mM and *V*_max_ of 2.88 µM/min. Recombinant enzyme showed pH optima at 6.3 and temperature optima at 34 °C. Asp gene was successfully cloned into *E. coli* BL21 which produced high level of asparaginase intracellularly with 85.25 % recovery of enzyme with a specific activity of 0.94 IU/mg protein. The enzyme was a tetramer with molecular weight of approximately 141 kDa.

## Introduction

l-Asparaginase enzymes (l-asparagine amidohydrolase) catalyse the hydrolysis of l-asparagine to l-aspartate and ammonia, and to a lesser extent, the hydrolysis of l-glutamine to l-glutamate (Ebrahiminezhad et al. 2011). Bacterial l-asparaginase are of two types: type 1 and type 2; type 2 showed antitumor activity because of which interest in l-asparaginase arose (Lee et al. 1989). Normal cells can synthesize l-asparaginase and because of their ability to produce this amino acid, normal cells are protected from l-asparaginase starvation unlike tumor cells which are dependent on an exogenous supply (Oza et al. 2011). Depletion of the circulating l-asparaginase by l-asparaginase enzyme results in anti-neoplastic activity. However, *Escherichia coli* and *Erwinia* sp. enzymes have been frequently used in cancer therapy (Avramis and Panosyan 2005).

l-Asparaginase from bacterial origin can cause hypersensitivity in the long term used, leading to allergic reactions and anaphylaxis. The toxicity is partially attributable to the glutaminase activity of these enzymes (Oza et al. 2011). l-Asparaginases with high asparaginase activity and negligible glutaminase activity are reported to be less troublesome during the course of antitumor therapy (Hawkins et al. 2004). The search for other asparaginase sources, with new immunological characteristics can lead to enzyme with less adverse effects. Furthermore, new studies have revealed potential application of this enzyme in prevention of acrylamide formation in fried potatoes and similar food products. Therefore, introduction of new fermentation and purification protocols for production of l-asparaginase II will be mandatory to satisfy these demands (Aghaeepoor et al. 2011). So, in this study we will be describing the cloning, expression, purification and characterization of recombinant l-asparaginase from *P. fluorescens* into *E. coli* BL21.

## Materials and methods

### Materials

l-Asparaginase-specific primers used in this study were synthesized by Sigma-Aldrich Co (India). Taq polymerase and dNTPs were procured from Bangalore Genei (India) Pvt. Ltd. and pET101 TOPO vector kit was obtained from Invitrogen (India). l-Asparagine and Lysozyme was obtained from Sisco Research Laboratories Pvt. Ltd, Mumbai. Sonicator used was manufactured by Qsonica and Fermenter was designed and manufactured by CFTRI. *E. coli* BL21, *E. coli* DH5α and *Pseudomonas fluorescens* isolates were precured from CSIR-CFTRI, Mysore.

### DNA isolation and synthesis

*Pseudomonas fluorescens* was inoculated into induced 50 ml broth and was grown overnight followed by centrifuging and washing thoroughly with sterile distilled water to remove all traces of broth and extraneous material. Extraction of DNA was carried out using Lysozyme solution. Sequences homologous to ASP were sought in the NCBI using BLAST. A DNA sequence of l-asparaginase from *P. fluorescens* (GI no: 255961261) was used for primer synthesis. PCR was used to amplify the full-length DNA. The DNA synthesis was carried out in a total volume of 25 µl, containing 1 µl of DNA templet, 1 µl each of both forward and reverse primer, 0.3 µl taq polymerase, 0.5 µl dNTP mix, 2.5 µl PCR Buffer and 18.7 µl milli q water. The PCR procedure comprised 30 cycles of 30 s at 94 °C, 1 min at 63.6 °C and 30 s at 72 °C. A final extension time at 72 °C for 10 min was performed after the 30 cycles. The resulting PCR amplicon was sequenced.

### Cloning of ASP gene

For cloning, the primers were designed in such a manner that they included the CACC sites at 5′ end of the PCR amplicon, so it can be used for TOPO PET101 vector. The forward primer PFVJF3: 5′-CACCATGACATGTGCTTTGAAGAGTTTCGTCCGG-3′ and the reverse primer PFVJR4: 5′-TCAGTATTCCCAGAACATCCGTTGCAGCTC-3′ was used. The resulting amplified PCR amplicon was obtained through PCR with the conditions mentioned above. The amplified product was cloned with the plasmid vector pET101. The cloned vector was transformed to competent *E. coli* DH5α cells. The transformed *E. coli* cells were grown into LB agar plate containing 100 µg/ml ampicillin, IPTG and X-gal. The recombinant clones were identified by blue/white selection and grown at 37 °C in 50 ml LB medium containing 100 µg/ml ampicillin. The plasmid was isolated from *E. coli* DH5α and was used to transform competent *E. coli* BL21 cells which were screened with blue/white selection.

### Pilot fermentation

The cells were grown into LB agar plate containing ampicillin. Scale up of recombinant clones was done for 6 L medium for fermentation in 10 L fermenter. The recombinant clones were grown in LB medium containing ampicillin (100 µg/ml) at fermentation conditions of 37 °C with agitation of 180 rpm and aeration of 2 vvm. Synthesis of asparaginase was induced by the addition of 1 mM IPTG after 6–8 h of growth.

### Purification of asparaginase

Purification of asparaginase was done 16 h after induction by IPTG, cells were harvested by centrifugation at 6000*g* for 3 h. Cells obtained after harvesting were resuspended into 15 mM phosphate buffer with pH 6.3, grounded manually with sterile sand and centrifuged at 10,000*g* for 15 min at 4 °C. The supernatant was collected and used for purification. The fusion protein purification was done by treatment with 2 mg/ml lysozyme for 2 h and then sonication at 60 % amplitude for 5 min with 30 s burst. After sonication, the extract was centrifuged at 10,000*g* for 30 min at 4 °C. The extracted solution was then loaded to a pre-equilibrated Sepharose 6B column (1 × 20 cm) with mobile phase 15 mM phosphate buffer with pH 6.3 (Gladilina et al. 2009). The fractions collected (2 ml) were assayed for asparaginase activity. The purified enzyme was dialyzed to remove salt.

### Estimation of ASNase activity

Asparaginase assay for native and modified ASNases was performed for the formation of β-aspartylhydroxamate (Ramakrishnan and Joseph 1996). Authentic β-aspartylhydroxamate was employed as a standard. One unit (U) of asparaginase is defined as the amount of enzyme that formed 1 µmol of β-aspartylhydroxamate in 1 min under assay conditions. The specific activity is expressed as U/mg/min of protein. Protein content was measured according to (Bradford 1976) using BSA as a standard.

### Kinetic analysis

#### Determination of kinetic constants

Steady-state kinetic measurements were performed to obtain kinetic constants *K*_m_ and *V*_max_, of the purified l-asparaginase were determined by the method of Lineweaver and Burk (1934) with different concentrations (0.5 mM–1 M) of l-asparagine as a substrate at room temperature using sodium phosphate buffer (15 mM, pH 6.3).

#### Effect of pH and temperature on enzyme activity

The effect of pH on purified enzyme was investigated in the pH range 5.0–9.0 (using 15 mM sodium acetate buffer for pH 5.0–6.0, sodium phosphate buffer for pH 7.0–8.0 and carbonate-bicarbonate buffer for pH 9.0). The enzyme was incubated in these buffers for 1 h and the assay was carried out as given above. The optimal temperature for l-asparaginase activity of the purified enzyme was measured by incubating the enzyme-substrate mixtures for 1 h at various temperatures (8–45 °C) at pH 6, and the liberated ammonia and β-aspartylhydroxamate were measured.

#### Effect of macro-nutrients on l-asparaginase activity

Macro-nutrients including sodium (NaCl), potassium (KCl), magnesium (MgCl_2_), iron (FeCl_3_) and calcium chloride (CaCl_2_) at 1 mM were evaluated for their effect on l-asparaginase activity by incubating the enzyme at optimum pH and temperature for 1 h, and then ASNase activity was assayed by estimating the product formed at 500 nm. Relative activities were calculated and compared with the native ASNase.

#### Thermal stability and half-life of cloned ASNase

To study the thermal stability of ASNase, the cloned enzyme was incubated at 28 °C (room temperature) and 37 °C in the absence of the substrate. At periodic intervals, aliquots were withdrawn and assay was performed as described above. The residual activity was expressed as percent of the initial activity. The inactivation rate constants (*k*_d_) were calculated from slopes of a semi-logarithmic plot of residual activity versus time, and apparent half lives were estimated using Eq. (1). The time where the residual activity reaches 50 % is known as the half-life (Kishore et al. 2014).

1 $$t_{1/2} = \, \ln \, \left( 2 \right)/k_{\text{d}}$$

## Results

### Cloning and expression of ASP gene

The DNA yield obtained through Lysozyme method from *Pseudomonas fluorescens* was of good quantity and suitable for further use. The DNA was used as a template for synthesizing and amplifying desired l-asparaginase DNA by PCR and the PCR amplicon was purified. To select the required expression product, pET101 vector was chosen. The amplified product was checked for the presence of gene of interest by using the desired primers by PCR. The fusion plasmid with gene was used to initially transform into *E. coli* DH5α. The plasmid from *E. coli* DH5α cells was purified and used to transform expression host *E. coli* BL21. Cell-free extract of the *E. coli* BL21 gave fusion protein-asparaginase complex with a total protein content of 2.16 mg protein/ml.

### Purification of asparaginase

**Fig. 1:**
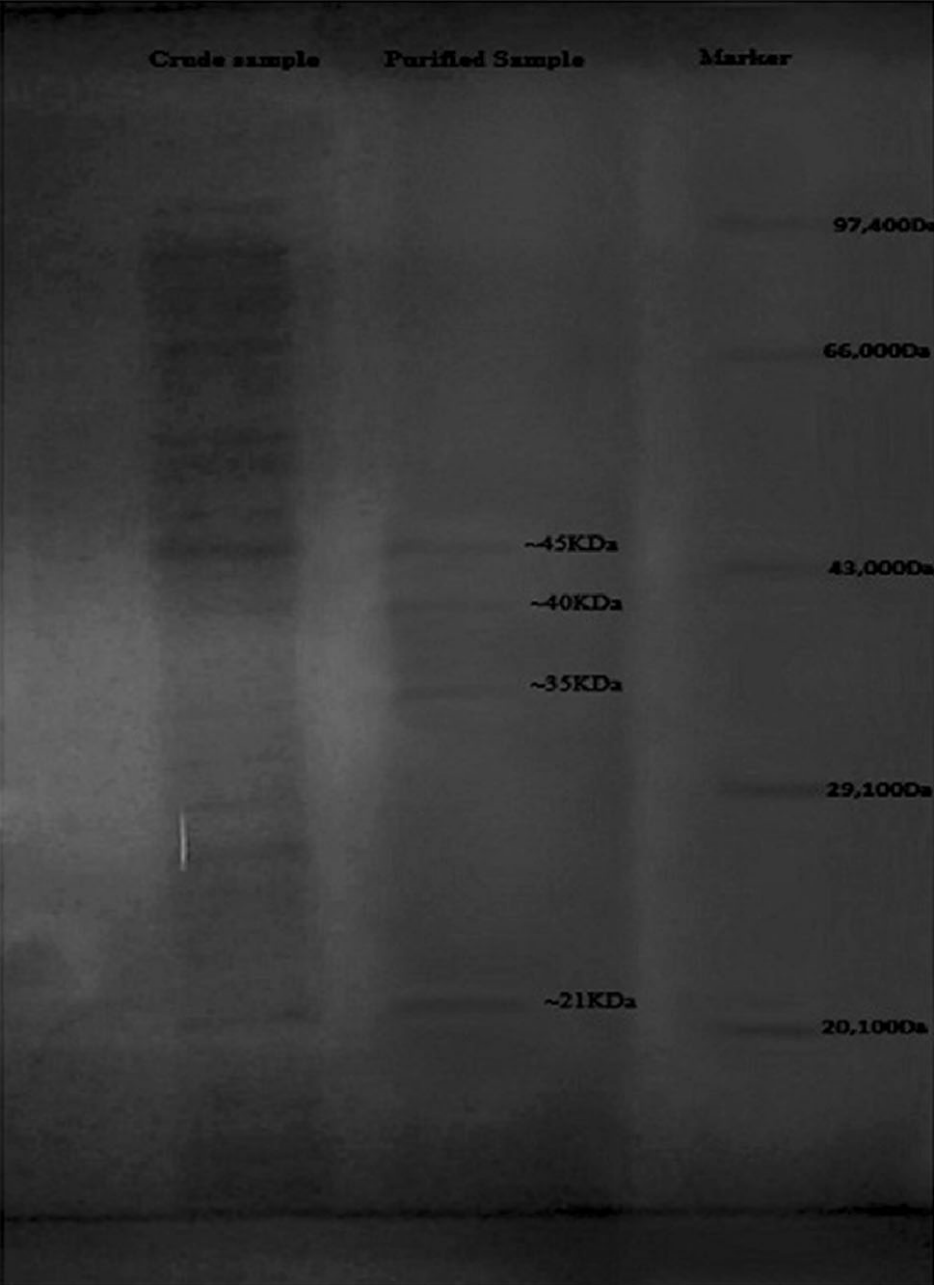
SDS-PAGE of recombinant l-asparaginase having crude sample, purified sample and Marker. l-Asparaginase was found to be a tetramer having 4 monomers of sizes 21, 35, 40 and 45 kDa after chromatographic steps of purification

Fusion protein-asparaginase complex was given a lysozyme treatment and sonication, and was then purified in a one-step procedure (Table 1). In the purification scheme, a Sepharose 6B column was used. Pre-treated enzyme extract was applied directly to the Sepharose column equilibrated with 15 mM phosphate buffer pH 6.3. The enzyme was obtained as per size exclusion and was eluted. The asparaginase activity was shown by fractions with a specific activity of 11.6 IU/mg protein. The purity of the final asparaginase was evaluated by SDS-PAGE, which showed that the Asparaginase is a tetramer with molecular weight of the cloned product around 141 kDa (Fig. 1).

**Table 1:** Purification of recombinant l-asparaginase from *E. coli*

Sample	Volume (ml)	Protein (mg)	Activity (IU)	Specific activity (IU/mg)	Fold purification	Recovery (%)
Cell-free extract	30	198	2.38	0.012	0	0
Lysozyme treated and sonicated	30	270	157	0.58	1	100
Sepharose column	300	140.88	133.84	0.95	1.64	85.25

*IU* is international units for enzyme activity

### Kinetic analysis results

#### Determination of kinetic constants

**Fig. 2:**
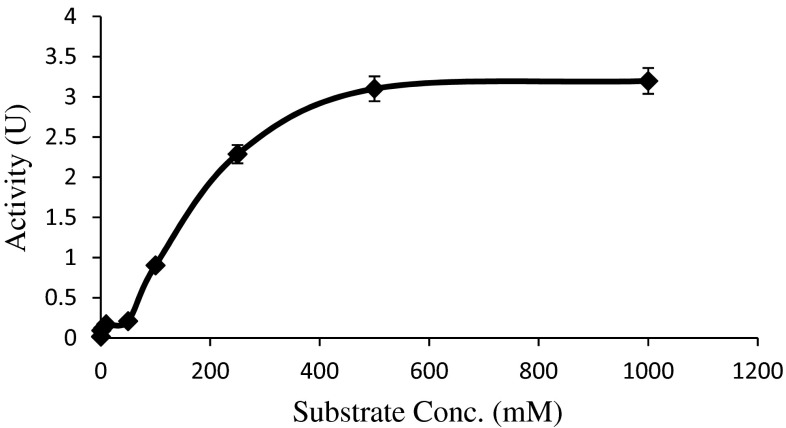
Michaelis–Menten kinetics of l-asparaginase versus the concentration of l-asparagine

Steady-state kinetic measurements were performed according to “Materials and methods”, Michaelis–Menten plot was done (Fig. 2) and kinetic constants *K*_m_ was found to be 109.99 mM and *V*_max_ was 2.88 µM/min.

#### Effect of pH on enzyme activity

**Fig. 3:**
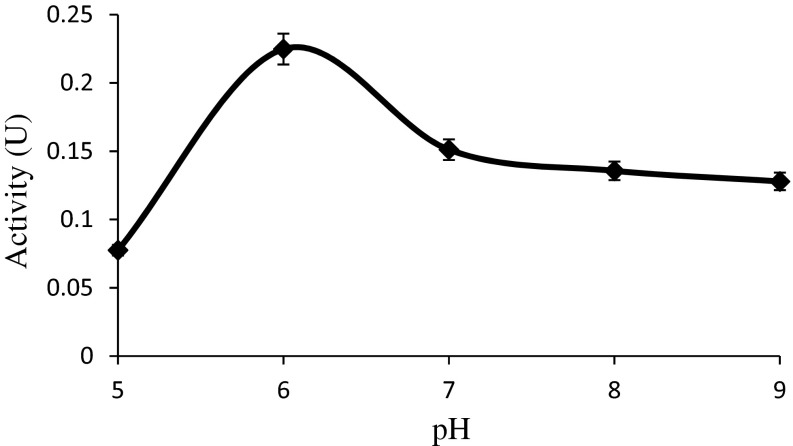
Effect of pH on l-asparaginase activity at room temperature

Since microorganisms are sensitive to the concentration of hydrogen ions present in the medium, pH is considered as an important factor that determines the growth, morphology and product formation. Effect of pH was studied in range of 5–9 according to “Materials and methods” and it was observed that the optimum pH was 6.3 (Fig. 3).

#### Effect of temperature on enzyme activity

**Fig. 4:**
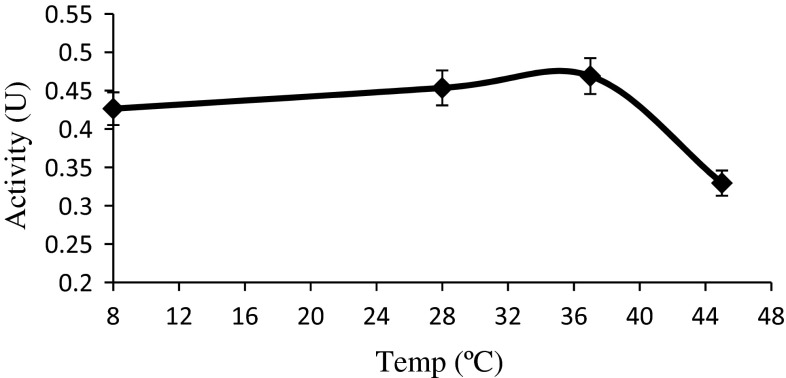
Effect of temperature on l-asparaginase activity at pH 6.3

After obtaining the optimum pH for enzyme, the experiments were run at phosphate buffer pH of 6.3 at different temperatures from 8 to 45 °C according to “Materials and methods” and it was observed that enzyme activities at 8 °C was low, and there was a gradual increase in activity with increase in temperature up to 34 °C which was found to be optimum temperature (Fig. 4). Further rise in temperature would have led to the degradation of enzyme and thermal deactivation; hence there is a decrease in enzyme activity.

#### Effect of metal ions

Effect of few metal ions was studied according to “Materials and methods”. With the cloned enzyme, not much inhibitory effect was seen on the enzyme activity (Table 2). The activity of control cloned enzyme was similar to the enzyme in the presence of metal ions.

**Table 2:** Inhibition of activities of enzyme with different Metal ions

Metal ions	Activity
Without inhibition	0.453
FeCl_3_	0.415
NaCl	0.438
KCl	0.395
MgCl_2_	0.422
CaCl_2_	0.430

Activity is expressed in terms of IU

#### Half-life of asparaginase

The enzyme assay was done and half-life was calculated according to “Materials and methods” at 28 °C (room temperature) and 37 °C (Table 3). Half-life of cloned enzyme was found to be 35 h at 28 °C and 45.5 h at 37 °C.

**Table 3:** Activities of l-asparaginase at different time interval at 28 and 37 °C

Time (h)	Activity at 28 °C (room temp.)	Activity at 37 °C
0	0.45	0.45
2	0.56	0.62
4	0.69	0.71
6	0.57	0.66
8	0.46	0.51
24	0.26	0.35

Activity is expressed in terms of IU

## Discussion

In the present study, we have produced l-asparaginase by cloning the asparaginase gene from *Pseudomonas sp.* into *E. coli,* for that we started our work with isolation of DNA, primer designing for asparaginase gene, cloning into pET101 for expression and pTZ57 R/T vector for sequencing, expression of the Asparaginase gene into *E. coli* BL21, obtaining large quantity of enzyme using 10 L fermenter, purifying the enzyme and finally characterizing the cloned purified Asparaginase was carried out. Recombinant l-asparaginase was developed by cloning l-asparaginase from *Erwinia carotovora* NCYC 1526 (Er A) and expressing in *E. coli*. The enzyme was purified by anion-exchange chromatography and affinity chromatography on immobilized asparagine (Kotzia and Labrou 2005). Recombinant l-asparaginase has been produced by the use of molecular cloning and genetic engineering techniques. *E. coli* mutants resistant to substrate l-asparaginase were studied by Spring et al. (1986). It was found that the genes encoding l-asparaginase-I and l-asparaginase-II were not sequence related.

Cloning of *E. coli* gene ansB encoding l-asparaginase-II, using strategy based on PCR, and sequencing the gene was discussed by Bonthron (1990). The amino acid sequence differed from 11 positions from the data previously derived by direct amino acid sequencing. Expression of l-asparaginase-II encoded by ansB in *Salmonella enterica* was found to be positively regulated by a cAMP receptor protein (cRP) and anaerobiosis (Jennings and Beacham 1993). Abundance of AS mRNA was measured by RQ-PCR as reported by Irino et al. (2007), the AS mRNA level paralleled the AS enzyme activity and the AS protein level. Cellular levels of AS synchronized with cellular resistance to l-asparaginase in cell lines. Recombinant l-asparaginase from *Erwinia carotovora*, and purified by 1-step chromatography, was described by Krasotkina et al. (2004). The kinetic properties showed that recombinant l-asparaginase combined the main advantages of *Erwinia chrysanthemi* and *E. coli*l-asparaginase-II. l-asparaginase from *Erwinia chrysanthemi* 3937 (Erl-ASNase) has been expressed in *E. coli* BL21 (DE3) pLysS (Kotzia and Labrou 2007). The enzyme was found to be a tetramer with molecular weight of approximately 141 kDa. It had a pH optima at 6.3 and temperature optima of 34 °C with *K*_m_ of 109.99 mM and *V*_max_ of 2.88 µM/min. Its activation energies were found to be dependent on the substrate. The l-asparaginase-II functional form exists as tetramer in *Pseudomonas geniculate* (Kitto et al. 1979) and *E. coli* (Khushoo et al. 2004) with molecular mass range from 140 to 160 kDa (Aghaiypour et al. 2001, El-Naggar et al. 2014). The l-asparaginase gene of *E. coli* alpha-acetyl actate decarboxylate gene (ALDC) of *B. brevis* were amplified by PCR and cloned into a new vector transformed into *S. cerevisiae*. Most of the enzyme activities were secreted into the medium and the new vectors had excellent segregation stability (Zhao et al. 2002). The enzymatic and structural properties of the recombinant enzyme were investigated and the kinetic parameters [*K*_m_, *K*_cat_] for a number of substrates were determined. The enzyme was later immobilized on epoxy-activated Sepharose CL-6B as described by Gladilina et al. (2009) who have taken SP-Sepharose (2.5 × 10 cm) equilibrated with 20 mM potassium phosphate buffer, pH 5.8. The immobilized enzyme retained most of its activity (60 %) and showed high stability at 4 °C. l-Asparaginase II gene isolated from thermotolerant *E. coli* strain, cloned in pET20b vector with 6His residues at a c-terminus downstream to the T7 promoter and pelB leader sequence, and biochemically characterized. Apart from clinical use, this enzyme can be used in fried starchy food preparation to reduce the acrylamide content, a potent carcinogen formed during the baking process by the reaction of asparaginase and sugar at hight temperature (Muharram et al. 2014). Application of microbial Lasparaginase in reducing acrylamide formation during the bread-making process, without affecting its physico-sensory properties and play an important role in reducing the formation of acrylamide. Reduction of toxic metabolites like acrylamide and HMF, i.e., Hydroxymethylfurfural (another toxic Maillard reaction compound) formed during baking, was also reduced by l-asparaginase treatment. The enzyme-treated bread was same as control bread with respect to organoleptic properties. This opens avenues for the application of microbial enzymes in obtaining safe foods (Mohan Kumar et al. 2014).

Cloning of *E. coli* gene ansB encoding l-asparaginase-II, using strategy based on PCR, and sequencing the gene was discussed by (Bonthron 1990). The amino acid sequence differed from 11 positions from the data previously derived by direct amino acid sequencing. Expression of l-asparaginase-II encoded by ansB in *Salmonella enterica* was found to be positively regulated by a cAMP receptor protein (cRP) and anaerobiosis (Jennings and Beacham 1993).

## Conclusion

The cloned enzyme was studied for its stability at 28 °C (room temperature) and at 37 °C, and it was observed that the cloned Asparaginase was stable with a half-life of 35 h at 28 °C (room temperature) and 45.5 h at 37 °C. Cloned Asparaginase was better in terms of activity and stability at 37 °C. This enzyme could be used/stored for longer time periods at room temperature for experiments relating to acrylamide reduction in food products. Also, its higher half-life at 37 °C makes it a more suitable enzyme to be used against cancerous cells.
